# Zonisamide Efficacy as Adjunctive Therapy in Children With Refractory Epilepsy

**Published:** 2013

**Authors:** Parvaneh KARIMZADEH, Mahmoud Reza ASHRAFI, Mohammad Kazem BAKHSHANDEH BALI, Mohammad Mahdi NASEHI, Seyedeh Mohadeseh TAHERI OTAGHSARA, Mohammad Mahdi TAGHDIRI, Mohammad GHOFRANI

**Affiliations:** 1 Professor of Pediatric Neurology, Pediatric Neurology Research Center, Shahid Beheshti University of Medical Sciences (SBMU), Tehran, Iran; 2 Professor of Pediatric Neurology, Department of Pediatric Neurology, Mofid Children Hospital, Faculty of Medicine, Shahid Beheshti University of Medical Sciences, Tehran, Iran; 3 Professor of Pediatric Neurology, Tehran University of Medical Sciences, (TUMS), Tehran, Iran; 4 Fellow of Pediatric Neurology, Pediatric Research Center, Shahid Beheshti University of Medical Sciences (SBMU), Tehran, Iran; 5 Assistant Professor of Pediatrics, Pediatric Neurology Research Center, Shahid Beheshti University of Medical Sciences (SBMU), Tehran, Iran; 6 General Physician, Brain and Spinal Injury Research Center, Neuroscience Institute, Tehran University of Medical Sciences, (TUMS), Tehran, Iran; 7 Associate Professor of PediatricNeurology, Pediatric Neurology Research Center, Shahid Beheshi University of, Medical Science (SBMU), Tehran, Iran

**Keywords:** Intractable Epilepsy, Antiepileptic drugs, Zonisamide

## Abstract

**Objective:**

Approximately one third of epileptic children do not achieve complete seizure improvement. Zonisamide is a new antiepileptic drug which is effective as adjunctive therapy in treatment of intractable partial seizures. The purpose of the current study was to evaluate the effectiveness, safety, and tolerability of Zonisamide in epileptic children.

**Materials & Methods:**

From November 2011 until October 2012, 68 children who referred to Children’s Medical Center and Mofid Children Hospital due to refractory epilepsy (failure of seizure control with the use of two or more anticonvulsant drugs) entered the study. The patients were treated with Zonisamide by dose of 2- 12 mg/kg daily in addition to the previous medication. We followed the children every three to four-weeks intervals based on daily frequency, severity and duration of seizures. During the follow-up equal and more than fifty percent reduction in seizure frequency or severity known as response to the drug.

**Results:**

In this study 68 patients were examined that 61 children reached the last stage.35 (57.4%) were male and 26 (42.6%) patients were female. After first and six months of Zonisamide administration daily seizure frequency decreased to 2.95±3.54 and 3.73±3.5 respectively. There was significant difference between seizure frequency in first and six month after Zonisamide toward initial attacks. After six months ZNS therapy a little side effects were created in 10 patients (16.4%) including stuttering(4.9%), decreased appetite (4.9%), hallucination (1.6%), dizziness(1.6%), blurred vision(1.6%) and suspiring(1.6%) as all of them eliminated later dosage reduction.

**Conclusion:**

This study conﬁrms the short term efﬁcacy and safety of Zonisamide in children with refractory epilepsies.

## Introduction

About 0.5 to 0.8% of children involved in epilepsy which is the most common cause of pediatric neurology clinics referral(1).Approximately eighty thousand new cases of epilepsy occur every year in children. Fortunately, many of epileptic children will recover spontaneously and gradually but severe complications develop in those with refractory seizure. Several studies show that about 10 to 30 percent of people with epilepsy, experiencing continuous seizures attacks as intractable epilepsy despite adequate anticonvulsant medications. Refractory epilepsy characterize lack of seizure control regardless of three or four best first-line drugs (as long as consumed in adequate dose and duration) (2-4) .Zonisamide (1,2 benisoxazol -3- methanesulfonamide) is a new generation anticonvulsant With broad spectrum antiepileptic activity(5,6).Zonisamide (ZNS) approved as monotherapy and add-on therapy of refractory partial epilepsies in adults and children. ZNS may be impressive in pediatric generalized seizures, particularly for myoclonic epilepsies.Furthermore, can be examined as a second-choice drug in the treatment of juvenile myoclonic epilepsy, infantile spasms and Lennox-Gastaut syndrome (LGS).Zonisamide is incomparable to other antiepileptic drugs(AEDs) due to chemical structure of non-arylamine sulphonamide and multiple modes of antiepileptic action(7-9). ZNS mechanisms of action include voltage related sodium and T-type calcium channel blockage (main pharmacologic effect), decline in glutamate dependent synaptic stimulation via potassium evoked responses blockade, enhanced gamma-aminobutyric acid (GABA) distribution from hippocampus, stabilizing neuronal membranes and suppressing neuronal hypersynchronization, dopaminergic and serotonergic comfort, erythrocyte carbonic anhydrase inhibition and destroying nitric oxide and hydroxyl radicals (10-14). ZNS represents a favourable pharmacokinetic index as absorbed quickly with peak plasma concentrations 2–6 h after oral administration and high bioavailability (95%) (15). ZNS has a long half-life of about 63 hours and take 1–2 weeks to get stable state after initiation the profile that facilitate once-daily usage (16). Researchers have not investigated Zonisamide efﬁcacy in Iranian children with various type of seizure in much detail. The aim of this study was to evaluate Zonisamide effectiveness in children with intractable epilepsy of different seizure types and it’s adverse effect characteristics.

## Material & Methods

From November 2011 until October 2012, 68 children referred to Children’s Medical Center and Mofid Children Hospital with refractory epilepsy entered the study. Inclusion criteria were children under 14 years and refractory epilepsy to previous anti-epileptic drugs (more than two conventional and new AEDs). Exclusion criteria were children with neurodegenerative diseases or hypersensitivity to anticonvulsants. All parents signed a written consent for study entry. Careful history containing type of seizure, seizure onset, developmental status, etiology of seizures, period of the treatment, type of antiepileptic drug administration and seizure frequency was taken from all patients. Seizures were confirmed and quantified by reports of parents, observation of videos that were detected by parents and direct observation in Hospital before and after treatment. Electroencephalography (EEG) was done for all patients. Diffused and continuous generalized paroxysmal epileptic discharges considered as severely abnormal EEG, when paroxysmal epileptic discharges covered more than 50% and 25% of EEG recording considered as moderate and mildly abnormal. Information from patient’s EEG and magnetic resonance imaging (MRI) findings were registered.Then, the patients were treated with ZNS by initial dose of 2 mg/kg daily in two or three divided doses in addition to the previous medication (more than two antiepileptic drugs / new or conventional/ concurrent AEDs are listed in table1). We followed the children every three to four-weeks intervals based on daily frequency, severity and duration of seizures. According to level of seizure response the drug doses were adjusted to maximum dose of 12 mg/kg daily.During the follow-up equal and more than fifty percent reduction in seizure frequency or severity known as response to the drug .Our clinical trial study confirmed by ethics Committee of Shahid Beheshti University of Medical Sciences. This study was registered in Iranian registry of clinical trial (IRCT) with number of IRCT2012091210508N2.

**Data Analysis**

Data were analyzed using SPSS 15 (SPSS Inc, Chicago, Illinios). P-value less than 0.05 was considered statistically different. 

## Results

**Fig 1:**
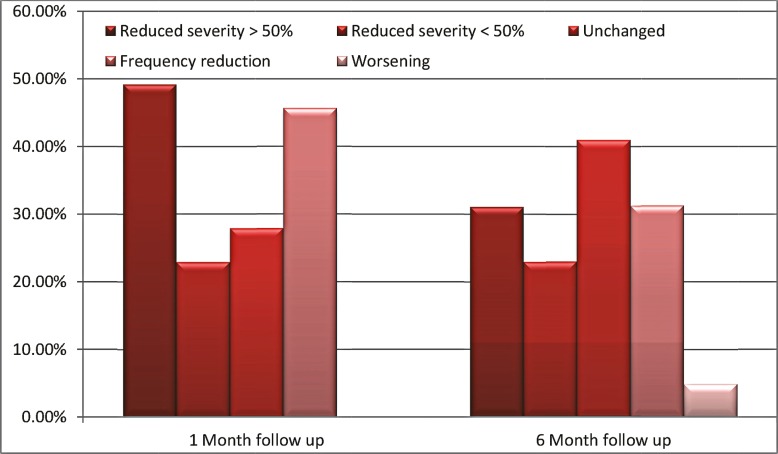
seizure reduction in the first and six-month follow-up

In this study 68 patients were examined that 61 children reached the last stage. Details of patient characteristics are listed in Table 1.Of these, 35 (57.4%) were male and 26 (42.6%) patients were female. Age range was from 1.5 months to 14 years with average of 73.9± 44.04 months. The age of seizure onset was from 3 days to11 years, and mean age at seizure onset was 27.1± 28.13 months. Daily frequency of seizures in patients varied from nine attacks every month (0.3 daily) to 50 attacks per day with average of 5.43 ± 7.21 seizures per day. The most common seizure form was generalized tonic clonic in 17 children (27.9%), partial in 16 patients (26.3%) (4 simple and 12 complex), and 11 patients (18%) with myoclonic seizure (Table 1).According to seizure etiology, 26(42.62%) were classified as idiopathic, 24 (39.34%) as cryptogenic and 11 patients (18%) as symptomatic epilepsy. Brain MRI of 19 patients (31.1%) were normal while the most common abnormality was brain atrophy in 17 children (27.9%)(Table 1). The EEG findings were normal, mild, moderate and severely abnormal in 4 (6.57%), 18 (29.5%), 24 (39.34% ) and 15 (24.6%) children respectively (Table1). Patients had used minimum 3 and maximum of 15 anti-seizure medications, the average number of 7.11± 2.97 prior this study. ZNS used at least 2 to maximum dose of 12 with mean of 10.4±1.27 mg/kg/day. After first and six months of ZNS administration daily seizure frequency decreased to 2.95±3.54 and 3.73±3.5 respectively. So within three and six months of this drug treatment patients daily attacks of seizure reduced up to 45.7% and 31.3% in turn. There was significant difference between seizure frequency in first (P=0.001) and six month (P=0.001) after ZNS toward initial attacks with no major discrepancy among three compared to six month seizure frequency(P=0.735).On basis of severity and duration of seizures in the first month follow-up, 17 patients (27.9%) had no change, 30 patients (49.2%) were responder (reduction of more than fifty percent) and 14 patients (22.9%) had reduction less than fifty percent(figure.1). The highest response to ZNS was in seizure type of infantile spasms (85.7%), generalized tonic clonic (58.8%) and myoclonic (54.5%) during the first month review. There was no significant correlation between the type of seizure and drug response in this time (p = 0.216). In six months follow up seizure severity and duration of 25(41%),19(31.1%),14(23.9%) and 3(4.9%) patients did not change, reduced more than fifty percent, had reduction lower than fifty percent and had worsened, respectively(figure.1). Myoclonic(54.5%),Infantile spasms (47.1%) and generalized tonic clonic (42.9%) were the seizure types with the highest response to ZNS in Six-month followup. The type of seizure had significant correlation with drug response in this time (p =0.069).None of the patients with mixed type of seizure respond to ZNS within first or six months review.Based on seizure severity and duration reduction there was no significant difference in the first month compared to six-month follow-up (p= 0.39). After six months ZNS therapy a little side effects were created in 10 patients (16.4%) including stuttering(4.9%), decreased appetite(4.9%), hallucination(1.6%), dizziness(1.6%), blurred vision(1.6%) and suspiring(1.6%) as all of them eliminated later dosage reduction. All of above complications observed with ZNS dose more equal 200 mg/day in patients over 6 years old and weighing more than twenty kilograms. 

**Table1:** Patient Characteristics

**Characteristic**	**Values**	**Characteristic**	**Values**
**Gender**		**Age**	
Male Female	35 (57.4%) 26 (42.6%)	Mean (SD) Range	73.9±44m 1.5m to 14 y
Seizure.onset		seizure frequency	
Mean (SD) Range	27.1±28.1 m 3 D - 11 y	Mean (SD) Range	5.43 ± 7.2 0.3 - 50
Seizure.type		MRI	
Tonic clonic Simple Partial Complex Partial Total Partial Infantile spasm Myoclonic Tonic Mixed	17 (27.9%) 4 (6.6%) 12 (19.7%) 16 (26.3%) 7 (11.5%) 11 (18%) 2 (3.3%) 8 (13.1%)	Atrophy PVL Tuberous sclerosis Migrational disorder Mesangial temporal sclerosis Cortical dysplasia Corpous callosum dysgenesis Basal ganglia lesion Focal lesion	17 (27.9%) 10 (19.4%) 3 (4.9%) 4 (6.6%) 2 (3.3%) 2 (3.3%) 2(3.3%) 1 (1.6%) 1 (1.6%)
Epilepsy.type		Failed AEDs prior to study	
Idiopathic Crypthogenic Sympthomathic	26(42.6%) 24 (39.3%) 11 (18%)	Mean (SD) Median Range	7.11 ±3 3 3-15
EEG.Quality		EEG.waves	
Normal Mild abnormal Moderate abnormal Severe abnormal	4 (6.6%) 18 (29.5%) 24 (39.34%) 15 (24.6% )	Spike HVSW Hypsarrhythmia Sharp wave Burst suppression	23(37.7%) 16(26.22%) 9(14.57%) 6(9.83%) 3(4.9%)
First month follow-up		Six-month follow-up	
Unchanged Improvement 75-99% reduction 50-75% reduction 25-50% reduction <25% reduction	17(27.9%) 9(14.8%) 8(13.1%) 13(21.3%) 8(13.1%) 6(9.1%)	Unchanged Improvement 75-99% reduction 50-75% reduction 25-50% reduction <25% reduction worsening	25(41%) 9(14.8%) 1(1.6%) 9(14.8%) 7(11.5%) 7(11.5%) 3(4.9%)

M= month, y= years, MRI=Magnetic resonance imaging, PVL=Periventricular leukomalacia, HVSW= High voltage slow waves

## Discussion

This study showed that Zonisamide reduced the daily seizure frequency as 38.5% and diminished severity and duration of seizure as more than fifty percent in 40.2% of patients. The findings of the current study are consistent with four clinical studies which recently performed by Brodie et al, Sackellares et al, Faught et al and Schmidt et al who reported responder rates up to 28-47% with ZNS (17). Also ZNS effectiveness declines the seizure frequency (more than fifty percent) in 48.7% of patients, reported by Giangennaro Coppola et al. This study confirmed our results (18).Stephen LJ has been showed ZNS effect on reducing seizure frequency in 39% of users about more than fifty percent Similar to decline rate of 30% reported by Hui Jeen Tan et al (19-20). Approximately 31% of patients who completed the Claudia B et al study continued to ZNS utilization compare to 24% determined by Yuen et al for pregabalin(21). In our study, ZNS reduction seizure frequency was different in six-month (31.1%) follow-up than the one month (49.2%). But the difference was not significant (Pvalue > 0.25). This finding is in agreement with Hui Jeen Tan et al findings which showed ZNS caused seizure diminution of 35.3% in two-month follow-up, also seizure had been reduced to 25.5% during twelve-month of follow-up(19). Loscher W et al has demonstrated that efficacy of anticonvulsant drugs decreased over time due to reduced drug receptor sensitivity leading to Pharmacodynamic functional tolerance that can even lead to complete loss of drug function (22).

ZNS caused seizure deterioration between 9.7% and 15.7% in study of Giangennaro Coppola et al and Hui Jeen Tan et al in turn reported exceed worsening rate of 4.9% (18-19). 

Infantile spasms (66.4%), myoclonic (54.5%) and generalized tonic clonic (50.9%) were the seizure types with the highest response to ZNS in our subjects with no effect on mixed type seizure. Consistent to the results of this study Giangennaro Coppola et al revealed the major ZNS efficacy on generalized seizures (57.4%) compared to partial types (37.1%)(18). 

As well Stephen LJ observed ZNS response on the control of generalized seizures (39%) more than the partial varieties (12.7%), versus the result reported by Hui Jeen Tan et al showed 23.5% in generalized similar to 25% for partial seizures (19-20) .Razieh fallah et al notified the most effect on myoclonic seizures up to 62.5%, furthermore contrary to our result showed that ZNS was effective in 43% of mixed type epilepsy(14). Giangennaro Coppola et al observed ZNS complication rate of 26.8% (greater than our report as 16.4%) that irritability and drowsiness were the most common complication in their study, all complications vanished with dose titrative deceleration (18).Hui Jeen Tan et al detected visual hallucinations in two patients that recovered by reduced the dose of ZNS as we reported (19). 

All of our patient’s complications occurred with high dose of ZNS dose (more than 200 mg/day in patients over 6 years old and weighting more than twenty kilograms). Stephen LJ declared that all 28.6% of his patients who have had ZNS complications taking more than 200 mg daily, which is similar to our result (20). 

So-Hee Eun et al demonstrated that high dose(6-8mg/ kg/day) of ZNS has the same efficacy like low dose(3-4mg/kg/day) but there was significant difference between the two groups in view of associated speech complications, this finding support our result about language stuttering via ZNS dose more 200mg/day. (23). 

**In conclusion, **this study conﬁrms the short term efﬁcacy and safety of ZNS in children with refractory Epilepsies. Future investigations with longer course of study are needed to evaluate ZNS prolonged effectiveness.
